# 0232. Evaluation of HFNC'S wash out effect; a comparison of open- and closed-mouth models

**DOI:** 10.1186/2197-425X-2-S1-P16

**Published:** 2014-09-26

**Authors:** N Nakamura, M Kurota, T Watanabe, Y Onodera, H Suzuki, M Nakane, K Kawamae

**Affiliations:** Yamagata Unversity Faculty of Medicine, Anesthesiology and Intensive Care Medicine, Yamagata, Japan

## Introduction

Although clinical studies of the high-flow nasal cannula (HFNC) and its effect on positive end-expiratory pressure (PEEP) have been performed, the mechanism of the washout effect and its relation with HFNC flow have not been well evaluated. Therefore, we made a respiratory model that can exhale with controllable end-tidal PCO_2_ (P_ET_CO_2_) to evaluate the washout effect of HFNC.Objective. To evaluate the quantitative results of HFNC's washout effect comparing open- and closed-mouth models.

## Methods

Optiflow^TM^ (Fisher and Paykel Healthcare, Auckland, NZ) was used as the HFNC system. The artificial respiratory model consisted of a lung model (the Dual Adult Training and Test Lung, Michigan Instruments Inc., Grand Rapids, MI, USA) and a ventilator (Puritan Bennett™ 840, Covidien, Dublin, Ireland). The HFNC and the respiratory model were connected by the airway model (Endotracheal Intubation Training Model LM-059, Koken Co., Ltd., Tokyo, Japan). Respiratory settings were as follows: respiratory rate, 16 breaths/min; inspiratory time, 1 second; and tidal volume (V_T_), 300, 500, or 800 mL. CO_2_ was infused into a distal site of the lung model to maintain P_ET_CO_2_, measured just below the glottis, at 40 mmHg at each V_T_ setting without HFNC. HFNC flow was changed from 10-60 L/min in each V_T_ setting, and the change of P_ET_CO_2_ was measured in the open- and closed-mouth models.

## Results

**Figure 1:**
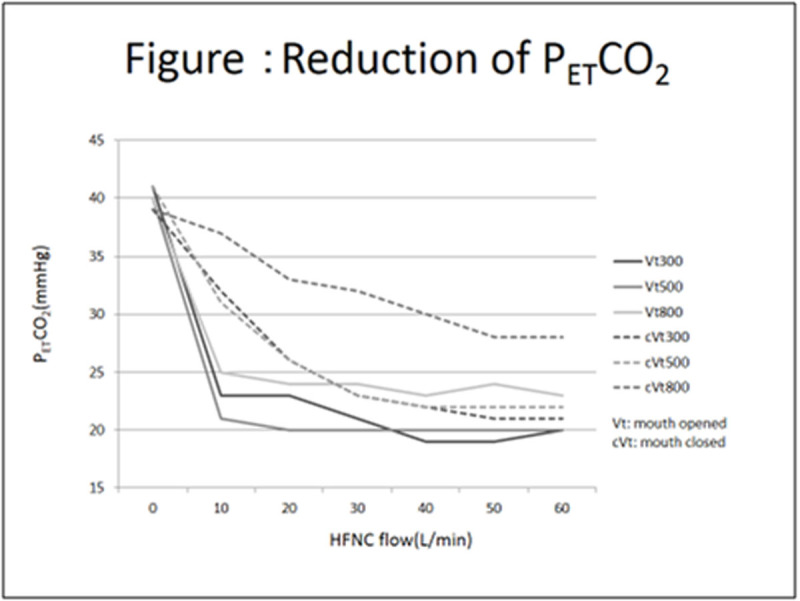
Reduction of P_ET_CO_2_

With any V_T_ setting in the open-mouth model, P_ET_CO_2_ quickly decreased to 20-25 mmHg as HFNC started at 10 L/min. Thereafter, P_ET_CO_2_ did not change with an increasing HFNC flow (Figure: solid lines). With the closed-mouth model, P_ET_CO2 gradually decreased as the HFNC flow was increased. The V_T_ settings of 300 and 500 mL had the same trends and reached the bottom level of 22 mmHg with HFNC flow over 50 L/min. The V_T_ setting of 800 mL had a smaller decrease in P_ET_CO_2_ to 28 mmHg (Figure 1: dotted lines).

## Discussion

Generation of PEEP by HFNC needs high flow as 35 L/min to generate PEEP of 3 cmH_2_O^1)^. In this study, it was demonstrated that HFNC's washout of the dead space is effective with relatively low flow as low as 10 L/min in open-mouth model. HFNC flow of 10 L/min can deliver gas of 166 mL/min, and this amount of gas delivery was thought to be enough to wash out the dead space during the exhalation time. The effect was weaker in the closed-mouth model, but by increasing the HFNC flow produced an adequate effect. In this closed-mouth model, more gas leaked from the nostril instead of the mouth, and therefore, less gas washed out the dead space, which caused a need for more HFNC flow to lower the P_ET_CO_2_.

## Conclusions

We concluded that the washout effect depends on HFNC flow especially with closed-mouth breathing while it may reach maximum with a relatively low flow of 10 L/min with open-mouth breathing.
